# Quantifying the Diversification of Hepatitis C Virus (HCV) during Primary Infection: Estimates of the In Vivo Mutation Rate

**DOI:** 10.1371/journal.ppat.1002881

**Published:** 2012-08-23

**Authors:** Ruy M. Ribeiro, Hui Li, Shuyi Wang, Mark B. Stoddard, Gerald H. Learn, Bette T. Korber, Tanmoy Bhattacharya, Jeremie Guedj, Erica H. Parrish, Beatrice H. Hahn, George M. Shaw, Alan S. Perelson

**Affiliations:** 1 Theoretical Division, Los Alamos National Laboratory, Los Alamos, New Mexico, United States of America; 2 Perelman School of Medicine, University of Pennsylvania, Philadelphia, Pennsylvania, United States of America; University of Texas at Austin, United States of America

## Abstract

Hepatitis C virus (HCV) is present in the host with multiple variants generated by its error prone RNA-dependent RNA polymerase. Little is known about the initial viral diversification and the viral life cycle processes that influence diversity. We studied the diversification of HCV during acute infection in 17 plasma donors, with frequent sampling early in infection. To analyze these data, we developed a new stochastic model of the HCV life cycle. We found that the accumulation of mutations is surprisingly slow: at 30 days, the viral population on average is still 46% identical to its transmitted viral genome. Fitting the model to the sequence data, we estimate the median *in vivo* viral mutation rate is 2.5×10^−5^ mutations per nucleotide per genome replication (range 1.6–6.2×10^−5^), about 5-fold lower than previous estimates. To confirm these results we analyzed the frequency of stop codons (N = 10) among all possible non-sense mutation targets (M = 898,335), and found a mutation rate of 2.8–3.2×10^−5^, consistent with the estimate from the dynamical model. The slow accumulation of mutations is consistent with slow turnover of infected cells and replication complexes within infected cells. This slow turnover is also inferred from the viral load kinetics. Our estimated mutation rate, which is similar to that of other RNA viruses (e.g., HIV and influenza), is also compatible with the accumulation of substitutions seen in HCV at the population level. Our model identifies the relevant processes (long-lived cells and slow turnover of replication complexes) and parameters involved in determining the rate of HCV diversification.

## Author Summary

Hepatitis C virus (HCV) is a RNA virus that infects over 170 million people across the world. It leads to a chronic infection in the majority of people who are infected (>70%). Most people only discover that they are infected long after initial infection. Thus, it is difficult to study the very early events in infection. Here we study 17 individuals during the earliest possible stages of infection, from before the virus is detectable in the plasma to around 35 days post-infection. We focus on understanding the viral kinetics and the diversification of HCV during this acute phase of infection. During chronic infection HCV is present in the host as a swarm of multiple variants generated by its error prone copying. We studied the early diversification of HCV during acute infection using a new mathematical model of HCV replication. We found that after a phase of fast increase in viral load, accompanied by viral diversification, there is a stabilization of viral load and diversity levels. Using our model, we were able to estimate for the first time the HCV mutation rate during acute infection. We estimated the median *in vivo* viral mutation rate is 2.5×10^−5^ mutations per nucleotide per genome replication (range 1.6–6.2×10^−5^), about 5-fold lower than previous estimates. We also used a different approach, based on results of classical genetics, to calculate HCV's mutation rate and obtained consistent results (2.8–3.2×10^−5^).

## Introduction

Hepatitis C virus (HCV) is a member of the hepacivirus genus within the flaviviridae family of virus, and it has a single positive stranded RNA molecule (∼9500 nucleotides) as its genome [1]–[3]. After entering a cell this RNA is translated into a single large polyprotein, which is cleaved to produce the viral structural and non-structural (NS) proteins [1]–[3]. The NS5B protein is a viral-specific polymerase, which is involved in replicating the HCV RNA genome [1], [4]. During genome replication the virion's positive strand RNA is copied into a complementary negative strand, which then must be copied back to produce a new positive strand. In the simplest replication model, this negative strand or a complex of the original positive strand and the newly created negative strand form an intermediate that acts as the template for producing new positive strands. This template plus various non-structural proteins form a structure called a replication complex [5]. If all new positive strands, and hence virions, are created from the same replication complex, we say that replication occurs by a “stamping machine" mechanism [6]–[9]. However, HCV infected cells often have more than one replication complex; indeed *in vitro* and *in situ* studies suggest there are about 40 such complexes in one infected cell [4], [10].

The HCV polymerase is an RNA-dependent RNA polymerase (RdRp) and hence does not possess error correcting mechanisms. Thus HCV replication, like that of other RNA viruses, is highly error prone [1]–[3]. Measuring the actual mutation rate, which derives both from the (+)RNA to (−)RNA and the (−)RNA to (+)RNA steps of replication, has been difficult [6], [11], [12]. A recent study determined the intrinsic error rate of the HCV polymerase *in vitro* using enzyme kinetic measurements [12]. They found high error rates, of ∼10^−3^ per site, for transitions and about 100-fold lower rates for transversions. Still, the *in vivo* mutation rate is likely different. Mutation is difficult to estimate *in vivo* due to selection, multiple rounds of replication and incomplete sampling [6], [11]. One proposed way to determine the *in vivo* mutation rate is to estimate it based on the frequency of lethal mutants in the viral population at any given time [13]. In fact, classical genetics shows that the frequency of a lethal mutation in a haploid population in mutation-selection balance is μ, the mutation rate. A recent study used this method to estimate an upper limit for the *in vivo* mutation rate of HCV as (1.15±0.29)×10^−4^ per nucleotide per replication round [13], which is within the range of other RNA viruses [6].

This high mutation rate is consistent with the high degree of HCV diversity found across the population of infected individuals [14], [15]. Indeed, HCV is highly variable, with multiple subtypes, and a global diversity that is higher than that of HIV-1 [15]. Clearly, this population level diversity, which reflects the HCV evolution rate, is in part prescribed by the mutation rate of the virus *in vivo* [16]. Moreover, in chronically infected individuals the HCV viral population is also diverse [17]. This diversity allows fast evolution and escape from immune [18] or antiviral drug pressure [19], and may contribute to HCV pathogenesis [18], [20].

An important question is how HCV diversity is generated. While it clearly depends on the mutation rate, we shall show using a model of HCV replication that it also depends on other parameters of the HCV life cycle [7]–[9], such as the long-lived nature of infected cells, as compared to HIV infected cells [21], [22], the existence of multiple replication complexes within an infected cell [4], [10], and the turnover rate of these replication complexes. In order to validate this model and obtain quantitative estimates of the *in vivo* HCV mutation rate, we shall exploit our observations in an accompanying report [23] and those of others [24], [25] that during the initial stages of primary infection the viral population is comprised of discrete low diversity lineages of viral sequences emanating from the transmitted/founder viral genomes [23]. Further, early on, diversity increases with time since infection. We shall show that the rate of diversification is not constant but rather slows as infection is established. Our model provides a quantitative explanation for this phenomenon. Analyses of HIV evolution in acute infection have been used to estimate the time since infection [26], [27]. Here, we know with reasonable accuracy the time of infection, but use the same ideas to estimate the *in vivo* mutation rate of HCV.

## Results

### In Primary Infection HCV RNA Levels Expand Quickly and then Plateau at a High Level

**Figure 1:**
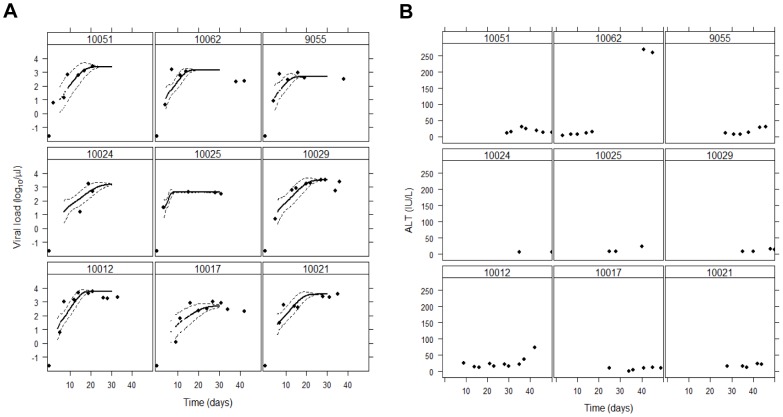
Profile of (A) viral load and (B) ALT in the subjects studied. The black symbols are the observed viral loads, the lines are the simulated trajectories with the model described in methods, and the dashed lines correspond to 95% CI based on 100 simulations. The parameters used for the simulations are given in Table 1. (The first week of increase in virus is very variable due to the stochastic nature of the process, and it is not represented in the graphs.). The profiles of ALT in (B) are about normal (the upper limit of normal – ULN – is ∼40 IU/ml [33], [34]) and much less than typical later in primary infection, where they can reach 10× to 20× the normal value [37].

The early dynamics of viral increase in HCV infection is different from that seen in other chronic infections, such as HIV [28] and HBV [29]. The HCV viral load in the subjects in this study increases roughly exponentially until it reaches a plateau (Figure 1A). This has also been observed in a prior study of acute HCV infection [30] and observed in chimpanzees experimentally infected with HCV [31]. Quantitative characteristics of this early increase are given in Table 1. The median time between the last negative sample and the first HCV positive sample in our dataset was 5 days, which is consistent with a viral dynamics analysis of larger numbers of plasma donors [30]. Because of this short interval, we assumed that the virus started expanding at the last negative sample. If the virus started expanding after this, our estimated expansion rate would be an underestimate. The median HCV RNA exponential growth rate was 2.2/day, corresponding to a doubling time of 0.31 days (or 7.4 hours). The median peak viral load observed was 3×10^6^ HCV RNA IU/ml and it took a median of 21 days to reach this level. The virus then stayed at approximately this high viral load level for a median of at least 26 days. In two subjects, we did not have enough follow-up to conclusively affirm whether a plateau exists or not. These estimates are in agreement with a previous study of 77 plasma donors with longer follow-ups, which reported an estimate of ∼6 days of viral expansion before the first positive measurement (compared to a median of 5 days in our dataset) and a mean plateau duration of ∼56 days [30].

**Table 1:** Kinetic and simulation parameters for each subject studied.

Subject	*r*	t_2_	VL_max_	Plateau	I_ss_	θ	*k*	μ	μ	μ
	(/day)	(days)	(log_10_/ml)	(days)	(%)			(Q1)	(Q2)	(5 h)
9055	2.4	0.29	5.93	31	1.8	0.019	0.75	N/A	N/A	4.5
10012	2.6	0.27	7.01	26	22	0.022	0.73	2.6	3.5	2.2
10017	2.0	0.35	5.98	26	2.1	0.014	0.89	5.3	3.8	3.9
10021	1.6	0.44	6.82	26	14	0.013	0.77	2.3	2.6	2.7
10024	1.2	0.59	6.47	N/A	6.4	0.008	0.86	2.6	1.9	2.5
10025	3.7	0.19	5.89	28	1.7	0.052	0.42	4.8	6.2	5.9
10029	1.8	0.39	6.77	23	13	0.014	0.82	2.2	2.4	2.3
10051	4.6	0.15	6.66	N/A	9.9	0.033	0.84	1.8	3.0	2.1
10062	2.2	0.31	6.42	38	5.7	0.018	0.75	3.8	1.6	2.3
**Median**	**2.2**	**0.31**	**6.47**	**26**	**6.4**	**0.018**	**0.77**	**2.6**	**2.8**	**2.5**
**Mean**	**2.4**	**0.33**	**6.44**	**28**	**8.5**	**0.021**	**0.75**	**3.2**	**3.1**	**3.1**
**Std Err**	**0.4**	**0.04**	**0.14**	**2**	**2.3**	**0.004**	**0.04**	**0.43**	**0.48**	**0.45**

*r* – exponential growth rate; t_2_ – doubling time; VL_max_ – maximum viral load; Plateau – time that the virus remains at the plateau; I_ss_ – percentage of cells infected at viral plateau, assuming that there are 10^11^ hepatocytes [40]; Q1/Q2/5 h – quarter 1, quarter 2 and 5′ half HCV genome, respectively. Other symbols described in text. The mutation rate is μ×10^−5^ per nucleotide per replication cycle.

The observation of the viral load plateau suggests that the number of infected cells reaches a steady state level a couple of weeks post infection. It is possible that this is a dynamic steady state, with removal of infected cells in equilibrium with generation of new infected cells. However, HCV is likely non-cytolytic [32], consistent with the normal levels of alanine aminotransferase (ALT<40 IU/L is upper limit of normal [33], [34]) in these individuals early in infection (Figure 1B). In addition, prior work has suggested that the cytolytic immune response takes weeks to months to emerge [31], [35], [36] (consistent with an increase in ALT to 10× to 20× the normal level late in acute infection [37]). Thus, it is likely that the rate of infected cell death during this early period is comparable to that of uninfected cells. The lifespan of uninfected hepatocytes has been estimated as being on the scale of months to years [38], [39], and thus infected cell death is probably negligible at these early times. In this case, the plateau in viral load suggests an equilibrium where all cells that can be infected are infected and producing virus. Assuming that there are 10^11^ hepatocytes in the liver [40], we estimate that a median of 6% (with range 1.7%–22%) of these are infected across our subjects (Table 1), consistent with experimental measurements in chronic infection [41], including recent estimates by two-photon microscopy of frozen sections of liver tissue [42]. Thus, primary HCV infection is characterized by fast growth of viral load to a plateau where only a minority of hepatocytes is infected.

### Dynamics of Early HCV Diversification

To evaluate how HCV diversity changes during primary infection, we performed single genome amplification (SGA) followed by direct amplicon sequencing [23], [26], otherwise known as single genome sequencing [43], at multiple time points in the subjects shown in Figure 1. SGA is achieved through serial dilution of the cDNA obtained by reverse transcription of HCV RNA from plasma (see Methods and [23] for details). We amplified 5′ half-genome sequences, on average 4879 nucleotides, covering core, E1, E2, p7, NS2 and most of the NS3 proteins of HCV. For early samples, with low viral loads, we amplified the same region, but in two separate assays of one quarter genome each to enhance sensitivity of amplification. In this way, we obtained 84 sets of sequences for the 9 subjects at multiple (between 3 and 5) time points. On average, we had 44 sequences per time point. All of the sequences were deposited in Genbank; see Li *et al.* [23] for further details and accession numbers.

We then aligned separately the set of sequences for each time point and for each sequence region and used a sequence visualization tool (Highlighter – www.HIV.lanl.gov), to analyze the sequence diversity based on individual nucleotides. This tool allowed us to identify low diversity monophyletic lineages corresponding to the putative transmitted/founder (T/F) viruses – the consensus at the earliest time point from SGA data [23]. We next confirmed that these lineages were maintained across the times sampled, to guarantee that we were analyzing the diversification of the same lineage over time. In cases where there were two or more putative T/F viruses, we analyze only the dominant lineage, as SGA sequence data was too limited to study the minor lineages.

From these 84 sequence alignments, we were able to study the evolution of the virus and the emergence of new mutations from very early in infection (mean: 7 days, range 2 to 15 days since the last negative sample across the 9 patients) until late in the plateau phase of viral load (mean: 33 days, range 21 to 42 days). We found that HCV sequence diversity increases quickly early on, but then stabilizes in 7 patients, starting at about day 14; in subject 10051 there was not enough follow up to assess this issue, and in subject 10029 a clear stabilization of diversity was not observed. The plateau of diversity occurred when an average of 46% of the sequences were still identical to the inferred T/F viral genomes. In three subjects (10029, 10062, 9055) there was an increase in diversity at late times, ∼35 days. Note that for 10062, this is coincident with an increase in ALT levels suggesting turnover of infected hepatocytes (Figure 1B).

We also found that in the vast majority of cases, HCV diversity at each time point was consistent with a star-like phylogeny, i.e. the viruses' sequences coalesce at a single genome founder [27], [44]. The only exception was the 5′-half of 9055 at the last sampling time point, day 38, when there was evidence for the onset of immune selection [23]. The mutations detected in the sequence sets also conformed to a Poisson distribution in the inter-sequence pairwise Hamming distances [27]. The exceptions were the 5′-half of 10029 at day 13, the second 5′ quarter of 10029 at day 34, the second 5′ quarter of 10051 at day 7, and the first quarter and 5′-half of 10051 at day 21. Due to the specifics of the HCV replication life-cycle, one predicts occasional violations in star-like diversification and in the fit to the Poisson distribution, because there is a non-negligible probability of shared stochastic mutations between HCV sequences. That is, shared mutations may occur even in the absence of selective forces. See the accompanying report [23] for a more detailed discussion of these issues.

### Model of HCV Replication during Primary Infection

We next developed a model of HCV replication to study the time course of accumulation of mutations and to estimate the *in vivo* mutation rate of HCV needed to describe the observations above. This stochastic model of HCV replication allowed us to study the time course of viral load changes and the accumulation of mutations in the study subjects (see Methods). In the model, we assume cells are infected by a single virion, i.e., that superinfection does not occur [45], [46]. We further assume that in every infected cell, on average, only a fraction *k* of newly synthesized viral (+) strand RNA (vRNA) is exported in new virions, and the rest, 1-*k*, forms new replication complexes (RC). We assume that vRNA degradation can be neglected, i.e., that the newly synthesized vRNA is either rapidly complexed with proteins and converted into stable RC, or rapidly encapsidated and exported. (Note that this is very different from analyses of HCV treatment, when production of vRNA and/or virion assembly/release may be blocked, and vRNA degradation becomes an important parameter in the clearance of infection [47]). These processes are assumed to continue until the cell generates a maximum number of replication complexes (*RC*_M_). Note that if we set *k* = 1, so that all synthesized vRNAs are exported, we recover the “stamping machine" mode of replication [7]–[9], where all virions result from the same replication complex, i.e., the same negative strand of RNA. The existence of multiple replication complexes within one cell corresponds to “geometric growth". In our model, after a virus is exported, a fraction 1-θ of the released virions is assumed to be cleared from circulation [5], and the remaining fraction, θ, is assumed to infect new cells. We also assume infected cells are long lived, and thus, we initially neglect death of infected cells during the first few weeks of infection. This assumption is consistent with the viral load profiles seen in the infected subjects, where viral load increases rapidly to a maximum level and plateaus at this level for weeks.

We used our model to reproduce the viral load data (Figure 1A). For each subject, the only free parameter available to determine the trajectory of virus over time is the fraction of vRNA exported, *k*, since all other parameters are fixed *a priori* or are calculated as a function of *k* (see Methods). We found that the model could describe the viral load data well with just this single adjustable parameter. The values estimated for *k* indicate that most of the synthesized vRNA is exported as virions (median *k* = 0.77, range 0.42–0.89). Moreover, the estimated values of *k* are quite similar among the different individuals, with the exception of 10025, who has a lower estimated *k* ( = 0.42). However, this subject has only one viral load measurement during the up-slope of the virus, which strongly influences the value estimated for *k*. Indeed, for this individual, choosing higher values for *k* lead to only slightly lower quality fits (not shown).

### The Mutation Rate of HCV In Vivo

Next, we used our model to analyze the diversification profiles of HCV in these patients. As the viral RNA is copied, errors in the incorporation of nucleotides are possible, i.e., mutations occur. If we let μ denote the probability that a base in the newly produced virion differs from that in the infecting virion, then for the stamping machine model the mutation rate, μ, is simply twice the rate at which bases are miscopied by the HCV RdRp, to account for the cycle of (+)RNA strand→(−)RNA strand→(+)RNA strand copying. With multiple replication complexes in a cell, opportunities exist for additional copying errors to be made since a newly synthesized (+)RNA strand needs to be copied again to make a replication complex. Every time a RNA strand incorporating a mutation is made, there is a probability that this mutation is lethal, and the virus or replication complex made from such RNA is non-functional. Prior experimental studies indicate that the fraction of random mutations that are lethal is about 40% in RNA viruses [48].

We incorporated mutation in our model to analyze the viral diversification data and estimate the mutation rate needed to match the observed accumulation of mutations. We assume that at time zero the putative T/F virus starts replicating and mutating. We then compute the decrease over time in the fraction of sequences identical to the T/F virus (i.e., “the fraction of unmutated viruses"). We compare this model prediction to the identical measurement in our subjects and varied the mutation rate to obtain the best agreement between model and sequence data obtained from plasma HCV RNA, which corresponds to (+)RNA strands.

**Figure 2:**
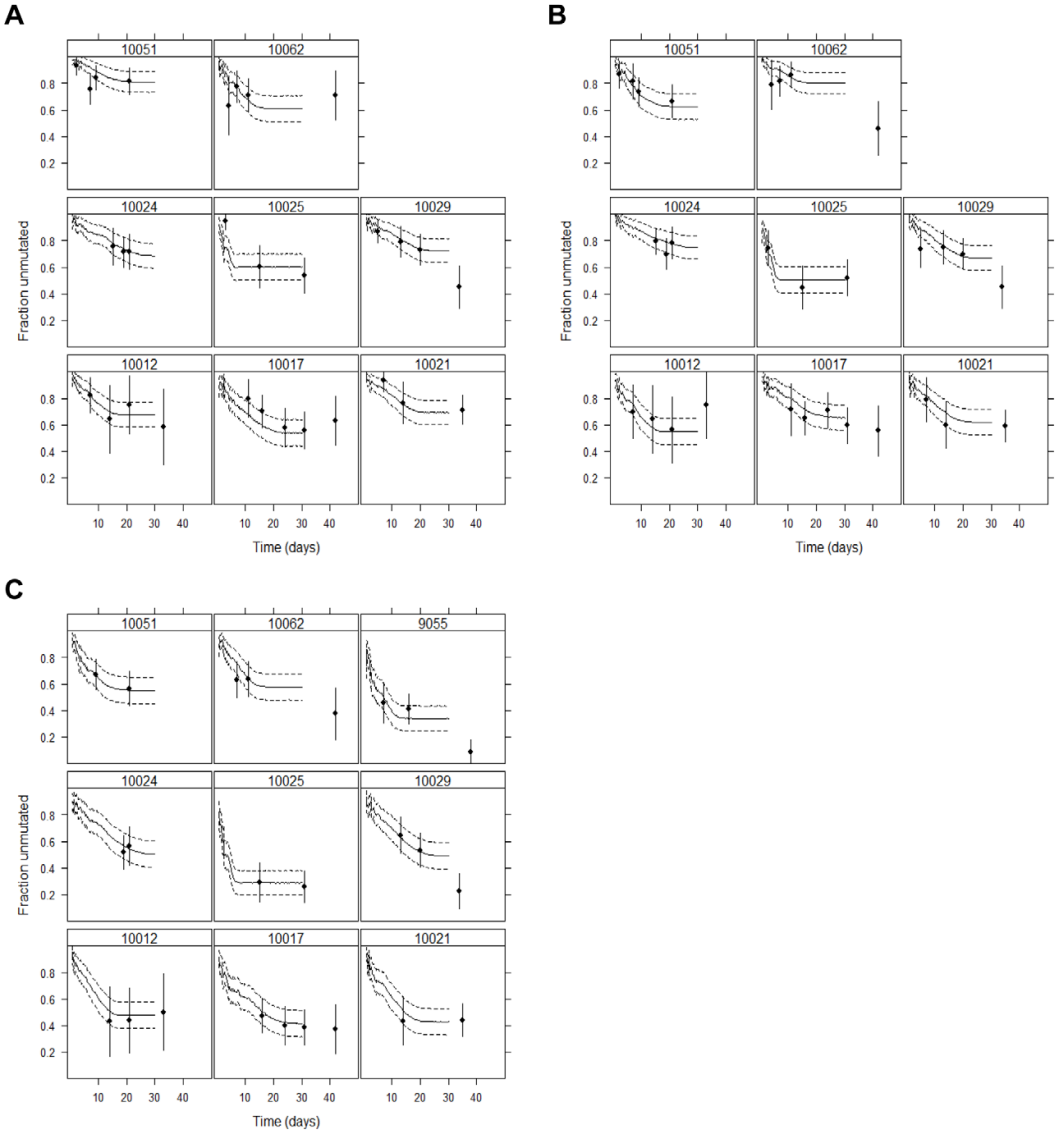
Fraction of sequences identical to the T/F virus over time. The symbols represent the SGA data and corresponding binomial 95% CI; the solid line is the average from 100 simulations and the dashed line the 95% CI for the proportion [64]. (A) data for the first quarter (Q1) of the 5′ HCV genome (note that for subject 9055, there is no data for Q1 or Q2); (B) data for the second quarter (Q2) of the 5′ HCV genome; (C) data for the 5′ half of the HCV genome.

The best description of the data was obtained for a median mutation rate (for the half-genomes) of μ = 2.5×10^−5^ per nucleotide per replication (Figure 2A–C). Moreover, this estimate was consistent across subjects and across regions of the genome (range: 1.6×10^−5^–6.2×10^−5^ per nucleotide per replication, Table 1).

Our model exhibits a fast decrease in sequence identity early in infection, as the viral load increases exponentially and more and more cells are infected, followed by a stable viral diversity level as the virus reaches and stays at its plateau. This stasis in viral diversification is compatible with the assumption that the plateau in viral load corresponds to a stable pool of infected cells. This indeed seems to be the case for 5 of the patients (Figure 2A–C); for 1 case there is not enough data. If the plateau in viral load corresponded to a dynamic steady state in which infected cells were dying and being rapidly replaced, our model would predict a continuous increase in diversification resulting from the continuous replacement of replication complexes. In a few cases, we did see an increase in diversity at times later than 30 days, and in three patients (10029, 10062, and 9055) the observed long term behavior (later than about day 35) deviates from that predicted by our simulations. This difference between model and data could be due to sampling error, for example the 95% CI for theory and data at day 42 overlap for patient 10062. Alternatively, some processes not accounted for in the model may be operational at these later time points, leading to increased diversity. For example, for subject 9055 anti-HCV antibodies are detectable at this late time point and there is strong evidence of CTL selection (escape or reversion) [23]; and for 10062 there is a late increase in ALT (Figure 1B), which suggests the initiation of a CTL response consistent with renewed cycles of infection.

**Figure 3:**
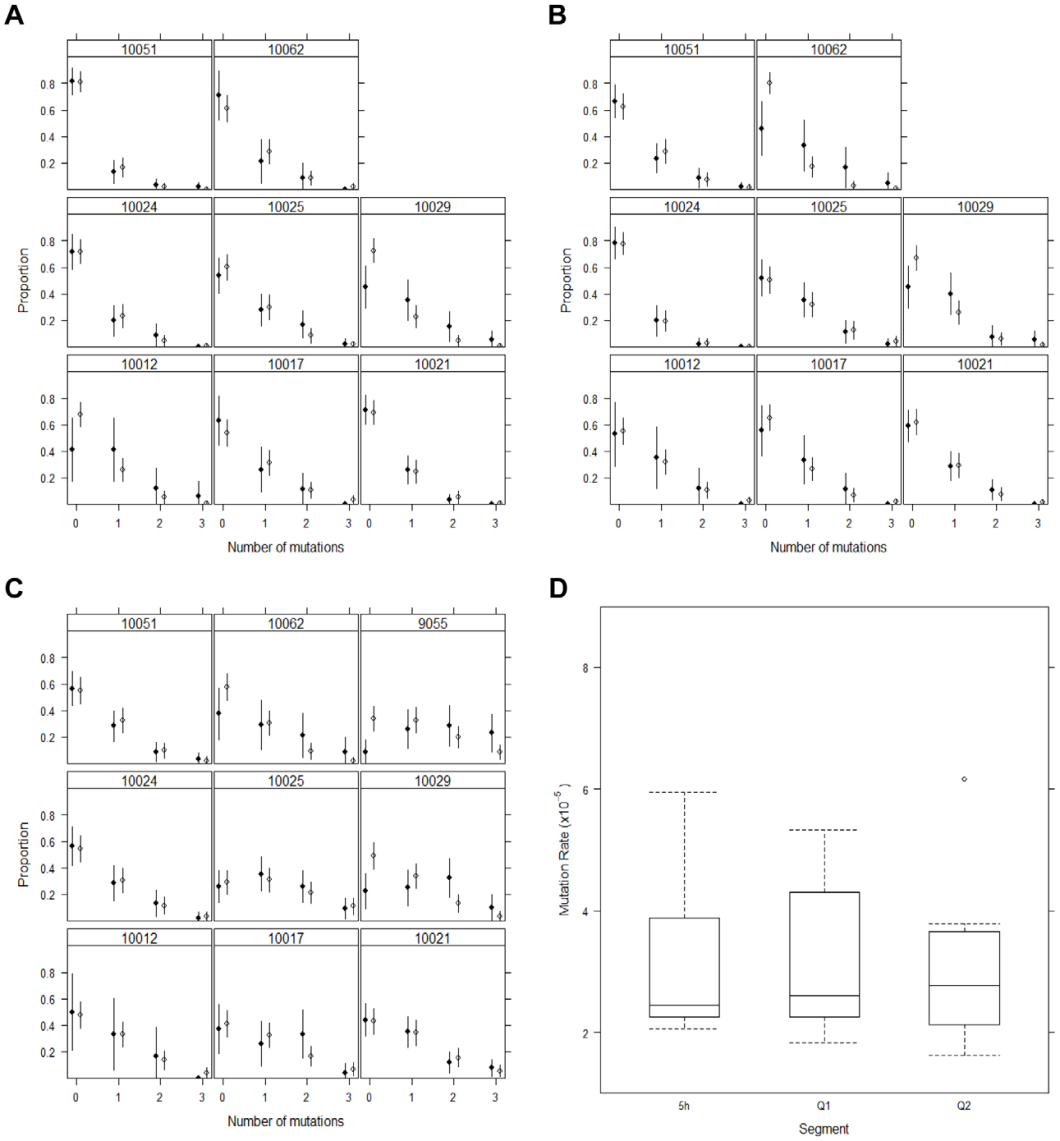
Spectrum of mutations in the data. The last sampling time (closed symbol) and the corresponding prediction by the model derived as the average of 100 simulations (open symbol) and respective 95% binomial CI based on the estimated mutation rates indicated in Table 1 and Figure 2 for each subject. (A) data for the first quarter (Q1) of the 5′ HCV genome (note that for subject 9055, there is no data for Q1 or Q2); (B) data for the second quarter (Q2) of the 5′ HCV genome; (C) data for the 5′ half of the HCV genome. (D) Summary box plot of estimated mutation rates for the different genomic segments.

Our model also makes predictions about the distribution of mutations across the population. Interestingly, our model not only matches the fraction of unmutated viruses, but also the fraction of viruses with 1, 2, 3, … mutations, even though this detailed data was not used to parameterize the model (Figure 3A–C). We obtained excellent agreement with the data, except when we observed a late increase in diversity in the three patients discussed above (10029, 10062, 9055). We tested this agreement for the 5 h genomes by a Monte Carlo test [49], since the number of expected mutations is low (<5) in several cases. The null hypothesis is that the data follows the theoretical expected values, and with the exception of those three patients, there was good agreement between observed and predicted mutation counts (p>0.05). Moreover, if we consider the distribution of mutations at the previous time for which we have SGA data, this agreement was also seen in 10029 and 10062 (p>0.05, and we cannot reject the null hypothesis).

We next tested whether our results were dependent on the particular values assigned to the parameters that we fixed in the simulation (see Methods). We found that both the viral load time course and the viral diversification were not sensitive to particular values of these parameters (Figure S1 in Text S1). For example, we assumed a maximum of *RC_M_* = 40 replication complexes per infected cell, as seen *in vitro* [4] and *in situ* [10]. Clearly this number could be different *in vivo*. However, our results were essentially the same, when we varied *RC_M_* from 10 to 80 (Figure S1 in Text S1).

To further confirm the robustness of our results, we next used the method suggested by Cuevas *et al.* [13] for estimating the mutation rate of HCV by analyzing the frequency of lethal mutations. Classical genetics shows that the frequency of lethal mutations is equal to the mutation rate, since all such mutations should be produced directly by mutation in the last replication round. As in Cuevas *et al.* [13], we used non-sense (stop codon) mutations as a proxy for lethal mutations. The concept is to count all stop codons in the data set and to divide this by the number of mutation targets (non-sense mutation targets – NSMT), i.e. codons that by a single mutation could generate a stop codon (see Text S1 for details). For these analyses, we were able to use all 17 patients in our cohort, thus expanding our data set.

In total we had 898,335 NSMTs and 13 stop codons in the over 1×10^7^ bases sequenced [23] (Tables S1 and S2 in Text S1). Surprisingly, 4 of the stop codons were identical and at the same position in 10051 at two different time points (see Table S1 in Text S1). This strongly indicates that this stop codon appeared only once in this patient, and that stop codons may not be lethal in HCV but instead complemented by intact genomes within the same cell. Thus, we counted this stop codon only once, for a total of 10 mutations leading to stop codons. A calculation identical to that proposed in [13] then shows that μ = 3.2×10^−5^ per nucleotide per replication, which is fully consistent with our estimate above. We also propose an improved way to calculate this rate from the same data (see Text S1), and with this method obtain μ = 2.8×10^−5^ (binomial 95% CI: 1.4–5.2×10^−5^).

Altogether, these data and analyses indicate that HCV sequences diversify early in infection, during the exponential increase of viral load, which is then followed by a plateau in diversity for up to a few weeks. The mutation rate needed to explain these observations (μ≈2.5–3.2×10^−5^ per nucleotide per replication, Figure 3D) is 5 and 100 times smaller than previously reported for HCV [13] and its purified RdRp [12], respectively.

We next investigated in detail why HCV diversification appears to stop after a few weeks of infection, and what processes could break this plateau in diversity, since in chronic HCV infection the virus is much more diverse [23]. In particular, we analyzed the effect of turnover of replication complexes and the emergence of the cytolytic immune response.

### Turnover of Replication Complexes

In the baseline simulations of the model, we neglected the turnover of replication complexes (RC). However, RC may degrade. In this case, to sustain viral replication, the RC would need to be continuously produced to balance their degradation. Thus, we next analyzed the impact on our model predictions of including RC degradation.

**Figure 4:**
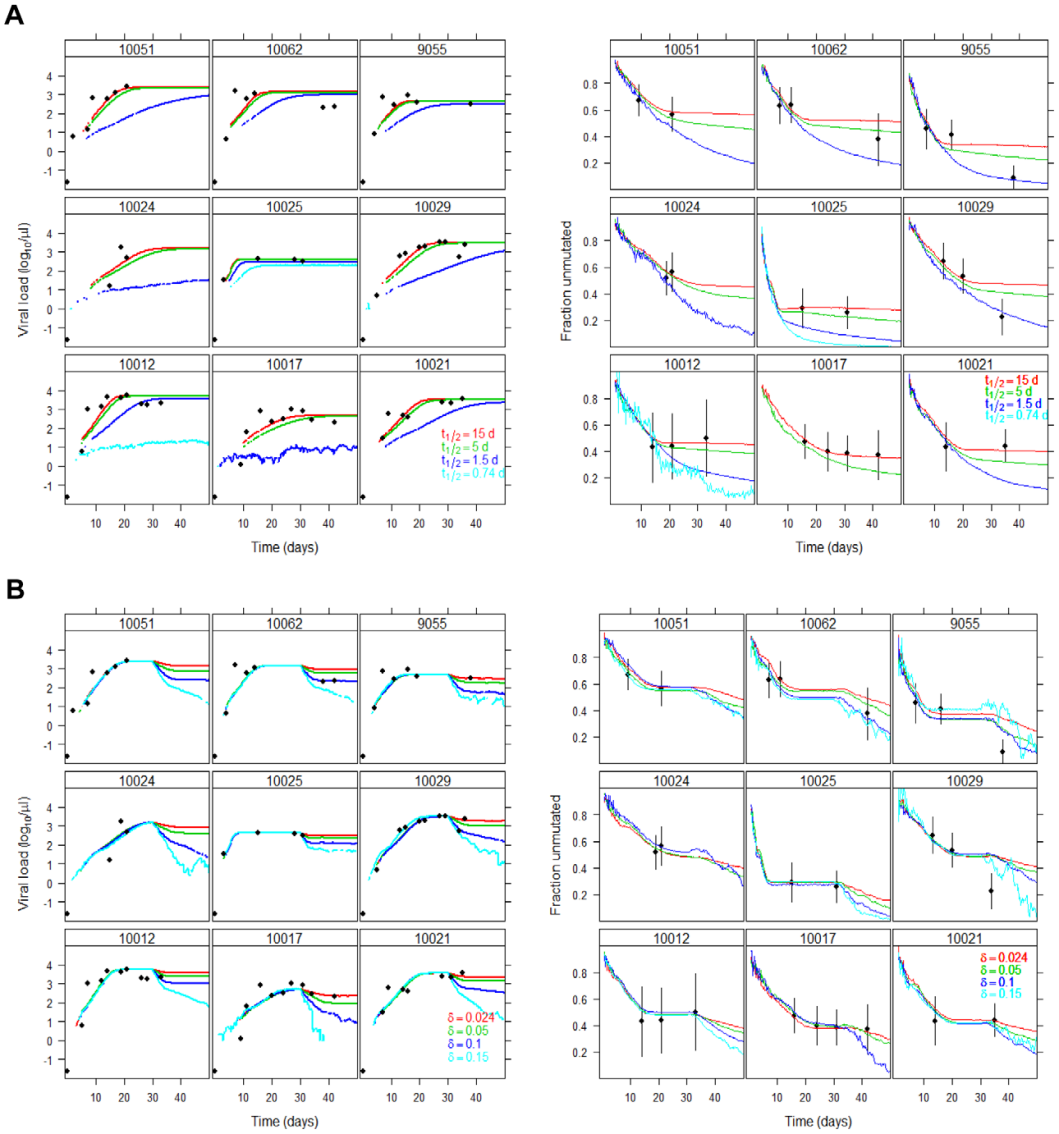
Changes in viral load and mutation profile predicted by the model. (A) different values for the half-life of replication complexes (ln 2/ρ), and (B) the emergence of a cytolytic immune response at 30 days post-infection. Note that if there were less than 5 runs leading to establishment of infection, no line is plotted, because the noise is too large. Thus in (A) for short half-life of replication complexes (i.e., *t*_1/2_ = 0.74 d in cyan) the line may not appear, because the infection was not established, or the line may disappear, because an initial infection was aborted.

For fast RC turnover (e.g., half-life 1.5 d), most (median of 59%) of the simulated infections die out, and those that lead to sustained infection show a slow growth of the virus that is not compatible with the data (Figure 4A, left panel). It is possible to recover fast viral growth rates, if one postulates that a larger fraction of newly synthesized RNA is used to form new replication complexes (i.e., if *k* is smaller). When the turnover of replication complexes is not negligible (t_½_<5 days), on the time scale of our simulations, the accumulation of mutations is faster at later times as replacement of replication complexes occurs (Figure 4A, right panel). In this case, to describe the data a smaller mutation rate would be needed, at least in some patients. Importantly, turnover of replication complexes also implies a continued increase in diversity throughout the observation period, since more (−) strand RNA needs to be made and hence there is more opportunity for mutations to occur. However, such a continued increase in diversity is not seen for subjects 10012, 10017, 10021 and 10025. On the other hand, this process could help explain the marked increase in diversity seen at late time points in subjects 10029, 10062 and 9055. Note however that even these subjects seem to have a stabilization of diversity prior to this marked increase, which is not compatible with fast turnover of replication complexes. If the turnover of replication complexes is much slower (eg., ∼15-day half-life) then the profiles do not differ from our baseline case where there is no turnover over the 50 day period studied.

Here we studied RC turnover inside the cell, but it is also possible that cells die due to the immune response against HCV, thus forcing re-generation of RC. Thus, we next considered the effects of cell turnover on the results of our model.

### Effect of the Immune Response

An effect of immune processes is removal of infected cells. Because there may be some limit to the number of infected cells in the liver [42], the death of infected cells may allow new cells to be infected, which in turn generates new RC and the opportunity for mutation accumulation. For all subjects for whom there is enough data, we find a stabilization of diversity, which in a few cases is then followed by a “sudden" marked increase at a later time point (10029, 10062, 9055). It could be that this latter pattern is an artifact of sampling. For example for 10062, the observed fraction of unmutated sequences at the three time points sampled have confidence intervals that overlap, and those fractions are not significantly different, p = 0.07 (Figure 2C, overlap of vertical bars). In our model this stabilization in diversity accumulation occurs because a steady-state is attained for the numbers of replication complexes and infected cells, without continued turnover. Rather than a sampling issue, it is possible that the observed increase in diversity is due to an immune response emerging at late time points, which leads to an increase of the infected cell death rate (δ). Indeed, this is indicated in studies of experimental infection of chimpanzees, where the immune response is delayed several weeks [31], [36]. In this context, an alternative explanation for the increase in diversity in 10062 is the appearance of such an immune response as suggested by the increase in ALT in this subject (Figure 1B). To study the effect of a late immune response that kills infected cells, we allowed for this process starting at 30 days post infection (Figure 4B). As expected, the emergence of an immune response lowers the viral load, possibly leading to a new lower viral load steady state, as is observed in some experimentally infected chimpanzees [31]. With the loss of infected cells, new cycles of infection occur along with creation of new replication complexes, and the model predicts a renewed increase in the accumulation of diversity, which mimics the data in some subjects (eg., 10062, 9055). However, we do not have enough data to precisely estimate the timing and magnitude of this immune response.

## Discussion

We analyzed the viral dynamics and viral diversification of HCV very early in acute infection. The early diversity of HCV is very low, and the inter-sequence Hamming distances follow a Poisson distribution, as would be expected when the mutations occur approximately at the same rate at all positions and the sequences are not selected for diversity [27], [44]. Given this observation, the number of mutations at early times should depend on the time since infection, the mutation rate and the biology of viral replication. This idea has been used before in the context of primary HIV infection to estimate the time of infection, assuming a given mutation rate [26], [27]. In the present study, the time of infection is known to within a short time window, with the first HCV positive sample within 5 days of the last negative sample. With this information, we could use our data to estimate the *in vivo* HCV mutation rate. By developing a model of HCV replication that takes into account the details of the viral lifecycle, we found the estimated mutation rate varied among subjects between 1.6×10^−5^–6.2×10^−5^ mutations per nucleotide per replication cycle, with a median of 2.5×10^−5^ (Table 1, 5 h genome). This estimate was very robust to different assumptions about model parameter values (see Text S1). Moreover, we systematically made conservative assumptions for the less well known parameter values leading to higher estimates for the mutation rate. To further confirm our results, we estimated the mutation rate by a completely different approach based on the frequency of stop codons (non-sense mutations), corrected by the number of non-sense mutation targets, as proposed by Cuevas *et al.* [13]. With this calculation we obtained a mutation rate of 2.8×10^−5^ or 3.2×10^−5^ mutations per nucleotide per replication cycle depending on the calculation method (see Text S1), which is consistent with the estimate from our more complex dynamical model and substantially less than the rate (∼10^−4^) estimated by Cuevas *et al.* [13]. A likely explanation for the difference between the findings of our nonsense mutation analysis and that of Cuevas *et al.* is that in our study *Taq* polymerase errors are eliminated from the finished sequences by the SGA-direct amplicon sequencing method and thus do not enter in the error rate calculations; this was not the case for the previous analyses [6], [13]. We further note that estimates of the HCV mutation rate based on nonsense mutations are likely to be overestimates since we found that stop codons were not always lethal (see Text S1). One explanation for this observation is that there are multiple HCV RNAs in an infected cell and another RNA may complement nonsense mutations. Indeed, we also found a case of a chronically infected patient who has a strain with a large deletion replicating in plasma at multiple time points [23]. Moreover, for dengue virus (in the same *Flaviviridae* family of HCV) there is a report of a viral strain with a stop codon that spread and attained a high frequency in the population, implying replication in both humans and mosquitoes [50].

In addition, our analysis does not account for mutational errors resulting from the cDNA synthesis step of the sequencing process, which again may lead to an overestimation of the mutation rate. However, we used Superscript IIITM Reverse Transcriptase (Cat. No. 18080-093, 2000 units, Invitrogen Life Technologies, Carlsbad, CA) that has been reported to have an error rate of ∼2×10^−6^ mutations/nucleotide/replication [23], [51], which is at least 10-fold lower than our HCV mutation rate estimates, and hence should not significantly influence our estimates.

Our estimates of the mutation rate for the HCV RdRp of ∼2.5×10^−5^ are notable because previous reports have suggested that the *in vivo* mutation rate of HCV is of the order of 10^−4^ mutations per nucleotide per replication [13]; and that the *in vitro* rate of the isolated RdRp could be as high as 10^−3^ [12]. One possible explanation for the latter discrepancy is that the mutation rates observed with purified RdRp enzymes are generally larger than those seen *in vivo*, because *in vitro* analyses cannot recapitulate the intracellular milieu of the replication or polymerase complex. For example, in the case of HIV reverse transcriptase, the errors measured with purified enzyme were found to be up to 20-fold higher than those measured in infected cells [52]. Another possibility is that we may have missed some low prevalence strains. However, a detailed power calculation shows that with the number of sequences obtained per patient, we would only miss strains that are present at very low levels, below 2% [23], which is much better than was possible before [25], [53] (see Li *et al.* [23] for a detailed discussion). Moreover, for the dynamical model we follow time courses and analyzed the fraction of virus identical to the T/F virus; and for the stop codon analyses, we corrected for the mutational targets. Both of these lower the impact of missing strains.

Given the low level of diversity observed in early infection and the relatively low mutation rate, the enormous diversity of HCV [14], [15], [18] and its high substitution rate (i.e., substitutions/site/year) have to be understood in light of HCV's replication mechanism [16]. Relatively long-lived infected cells, with multiple replication complexes allow for the accumulation of diversity in the virions produced. At the same time, the turnover of both replication complexes and infected cells, which must surely ensue as the immune response develops, allows for renewed generation of diversity throughout the course of infection (compare 10062 in Figure 1B and Figure 2C). Indeed, it could be that these details of the life cycle are responsible for the large diversity of HCV. We note that HIV and influenza, which are thought to have similar mutation rates to the one estimated here [6], [52], also have high substitution rates [54]. In this context, we see that accumulation of diversity is not only dependent on mutation rate, but also to a great extent on the particular processes of the viral life cycle [7], [8], [16]. Clearly, the pressure of the immune response, once established, will be important in determining relative fitness of many of the mutations and in determining the spectrum of mutations observed. That we see only scarce evidence of positive selection in our dataset indicates that there is a window of several weeks before the effects of the immune response can be detected.

Another important parameter that we estimated was the fraction of infected cells during the early plateau in viral load, which ranged between 1.7% and 22% of hepatocytes. This fraction is in reasonable agreement with other studies of HCV [41], [42]. In our model, this fraction depends on the value assumed for the maximum number of replication complexes (*RC*_M_). The larger the number of replication complexes in an infected cell, the more viruses this cell can produce per unit of time, and thus the fewer the number of infected cells needed to maintain a given steady state viral load. However, increasing *RC_M_* has little effect on our estimate of the mutation rate (see Text S1).

In this study, we constructed a simple model of HCV replication that tried to capture the most salient features of the viral life cycle. Moreover, we were careful to choose parameters consistent with the literature *a priori*, so that only 2 parameters had to be adjusted to fit the data on viral growth and diversity increase. We tested variation in the model assumptions and found that the results were quite robust. Still, it is clear that many complexities could be added to the model. For example, instead of having a fixed *RC*_M_, we could allow it to vary from cell to cell and possibly even from time to time; or we could allow for a distribution of generation times for RNA synthesis. These and other processes are easy to include in the model, however we opted to keep to the essential aspects of the replication process, so that we did not have to make further assumptions, which would complicate the interpretation of the results. In essence, this is akin to choosing a simple experimental system that is amenable to easy manipulation and interpretation of results, even if it does not represent fully all the details of *in vivo* system.

Altogether, the unique dataset presented here, including HCV viral kinetics and genomic diversification very early in infection, revealed that the initial exponential expansion of HCV RNA is followed by a plateau in viral load that lasts up to a few weeks [30]. The initial viral expansion is accompanied by a fast early increase in sequence diversity, whereas during the viral plateau viral diversity remains approximately constant. During the plateau viral production continues but is simply balanced by the rate of viral clearance. In order to understand why viral diversity did not continue to increase during this period, we develop a novel stochastic model of HCV infection. The basic idea behind the model is that during the early exponential expansion of the virus, new cells are being infected and generating multiple replication complexes in each infected cell. This involves multiple copying events of (+)RNA to (−)RNA to (+)RNA, etc, with errors potentially being generated at each stage. We postulate that once the viral plateau is reached a stable population of long-lived infected cells has been generated which then produce the plateau virus without any need for new RC generation. If no new replication templates are made then there is little opportunity for mutations to accumulate, though each virus can still mutate in relation to its parent RC due to the (−)RNA to (+)RNA copying event. We found that our model, based on this idea, agreed with both the viral load kinetic data and the sequence diversity data if we assumed that the *in vivo* mutation rate of HCV is ∼2.5×10^−5^ per nucleotide per replication cycle. This is about 5-fold lower than previously reported, but still high enough that coupled with the long-lasting nature of HCV infection and the very high turnover of virus in chronic infection leads to substantial HCV diversity in an individual and in the population.

## Materials and Methods

### Patient Population

Plasma samples were obtained from seventeen regular source plasma donors, who became HCV infected during periods of twice-weekly plasma donations. The donors were untreated and asymptomatic throughout the collection period. All subjects gave written, informed consent and the study protocols were approved by institutional review boards at the University of Pennsylvania, the University of Alabama at Birmingham and Duke University. HCV RNA and antibodies were analyzed as described elsewhere [23].

### Single Genome Sequencing

Single genome amplification (SGA) followed by direct amplicon sequencing was performed on sequential plasma vRNA samples (i.e., (+) RNA strands), as described in detail elsewhere [23].

For our dynamical analyses, we selected subjects who had at least two time points sampled with single genome amplification assays [23]. Thus, three subjects were not included – 6213, 6222, 10004. Six subjects (10002, 10003, 10016, 10020, 10029, 106889) had more than 7 putative T/F viruses, which makes a diversification analysis impractical, both due to the complexity of the viral species in the subjects and the small number of sequences representing each lineage [23]. The exception was 10029, who had a dominant lineage with more than 38 sequences for each time point, and we included this subject in our analyses. Thus, there were 9 subjects who were sampled at multiple time points and who had a clearly dominant putative T/F virus lineage [23]. Here we only analyzed these dominant lineages, for which we have the most data (SGA sequences).

### Sequence Analysis

Sequence alignments were initially made with ClustalW and then checked individually using JalView 2.6.1 (www.jalview.org). We used ConsensusMaker (www.HIV.lanl.gov) to calculate the consensus of the first set of sequences sampled by SGA, which is the putative T/F virus [23]. The set of sequences from each SGA sample with the corresponding consensus was analyzed by PoissonFitter (www.HIV.lanl.gov) to calculate for each sequence the number of mutations away (i.e., Hamming distance) from the T/F, and to test whether sequence diversification conforms to a star-phylogeny and if the set of inter-sequence Hamming distances follow a Poisson distribution [44].

Altogether we analyzed time courses of thousands of sequences with over 11.9 million base pairs and 1887 mutations [23].

### Model

To analyze the process of replication of HCV and how it affects the generation of diversity in primary infection, we developed an agent based model of HCV infection and replication. We assumed cells are infected by a single virion, and that in every infected cell, on average a fraction *k* of newly synthesized viral RNA (vRNA) is exported in new virions, and the rest, 1-*k*, form new replication complexes in the cell. These processes continue until the cell contains a maximum number of replication complexes (*RC*_M_). We assume this maximum value is set by the availability of host factors. After a virus is exported, a fraction 1-θ of released virions are cleared from circulation, and the rest, θ, infect new cells.

As the vRNA is copied, errors in the incorporation of nucleotides are possible. Every time a mutation occurs, there is a probability that this mutation is lethal, implying a virus or replication complex made using such mutant vRNA is non-viable. Sanjuan [48] estimates that the fraction of random mutations that are lethal is about 40% for RNA viruses.

We assumed HCV is noncytolytic [32]. Thus, infected cells can produce virus for long periods of time – until the infected cell dies, either from natural death or immune attack. Early in acute infection there is little evidence of cytotoxic T cell activity and CD8+ T cells do not appear to enter the liver until many weeks after infection [36]. In addition, normal hepatocytes live for months [39] to a year or more [38], thus, we either totally neglect death of infected cells or allow death after the first few weeks of infection. The assumption of no early death is consistent with the normal levels of alanine aminotransferase (ALT) measured in these individuals (Figure 1B) and the viral load profiles, where viral load increases rapidly to a maximum level and then stays at that level for some time. (This is in stark contrast for example with HIV, a cytolytic virus, where a clear peak in viral load is seen during primary infection followed by a decrease in viral load [28].)

Replication of the RNA and formation of a new virion or replication complexes is not instantaneous, as it takes a certain amount of time for synthesis of the different molecular components and their assembly. Although this time is most likely variable from replication cycle to replication cycle and from cell to cell, we assume that it is similar for all replication events in our model, fixing it at an average time to complete all the replication steps. This time we call the “generation time". Most likely it will take longer to produce the first copied RNA upon cell infection than later ones, as various molecular events need to occur before virus production begins (eg., uncoating, polyprotein synthesis and cleavage, assembly of the replication complex, etc) [5].

In the simulation, based on the assumptions described above, we follow the number, age (in the sense of generations) and mutational burden of each virion and each replication complex inside infected cells. The simulation was implemented in the R language (www.r-project.org). Because these are stochastic simulations, there is variability from one run to the next, even when all parameters remain the same. Thus, for each patient and each set of parameters (in Figures 1–4) we present results from 100 runs. Including more runs (we tested some cases with 200 runs) does not significantly alter the results presented.

### Model Parameters

The parameters of the stochastic model are as follows:

#### Generation time

During stable cell infection and virus production, we assume that it takes ∼6 h for a cycle of replication to produce new virions or replication complexes based on the following argument. A study of HCV replication kinetics [4] found that there are about 200 (+)RNA strands in a cell at steady state. With 40 replication complexes per cell, if we assume at steady state (+)RNA is being produced at rate α and degraded by a first-order process at rate *d_R_*, then at steady state 200 = α/*d_R_*. In treatment experiments in the replicon system the half-life of (+)RNA was found to be between 11 and 18 h [55]–[57], i.e. ∼15 h, so that *d_R_* = 0.693/15 = 0.0462 h^−1^ and α = 9.24 h^−1^. Then with 40 replication complexes per cell, each one would be producing (+)RNA at a rate of 9.24 h^−1^/40 = 0.231 h^−1^ and it would take 1/0.231 h^−1^ = 4.33 h to produce one new (+)RNA. Allowing for some extra time for assembly of a virion or a new replication complex, we thus assume ∼6 h for a cycle of replication.

#### RC_M_ – maximum number of replication complexes in an infected cell

Experimental results show that about 40 replication complexes can exist in one infected cell [4], [10]. Our baseline results use this number, but we also vary this parameter.

#### ρ – turnover of replication complexes

It has been observed after introducing treatment in a replicon system, that the half-life of (−)RNA was ∼12 h [56], but this decay only started after a 12 h delay. In our simulation, this would correspond to a degradation probability for the replication complex of ∼0.3 per generation. To see this, note that with a half-life of 12 h, a (−)RNA, or we assume equivalently a RC, decays at an average rate *d_RC_* = 0.693/12 h = 0.0577 h^−1^. To convert a continuous rate to a probability that an event occurs during a time interval Δt , note that by the exponential distribution the probability that a RC that is decaying with an average rate d_RC_ degrades at or before a time Δt has elapsed is given by 1- exp(-d_RC_ Δt) [58]. Choosing Δt = 6 h, i.e. a generation and *d_RC_* = 0.057 h^−1^, the probability of degrading in one generation is ρ∼0.3. However, we expect this to be an upper limit for ρ, the probability of degradation in the absence of treatment, because replication complexes are protected within vesicular membranous structures (VMS) adjacent to the ER membrane [59]. Indeed, the 12 h delay until the start of degradation of (−)RNA, which is thought to be mainly localized within the VMS, supports this idea. Initially, as a conservative approach to estimate the maximum mutation rate, we will assume that replication complexes are not degraded on the time-scales involved in primary infection, i.e., ρ = 0. We later allow RC degradation (ρ>0) and ask what impact it has in the dynamics of virus and viral diversity.

#### *k* – probability that a newly formed vRNA is exported as a virion

For each subject, we choose *k* to match the observed viral load profile. We varied *k* between 0 and 1 in increments of 0.01 and found the value that best describes the data by minimizing the sum of squared residuals, i.e. the difference between model and viral load data. For each case, we ran 100 simulations and then chose the value of *k* that led to the best match of the average of the viral load in the simulations with the observed viral load.

#### δ – turnover of infected cells

Hepatocytes are in general long lived cells, but infected cells may die faster due to viral effects. However, HCV is thought to be non-cytolytic [32], thus, we assume that the infected cell death rate is similar to that of uninfected hepatocytes, and can be neglected (δ = 0) in the time frame of our study. We also investigate the effect of the emergence of the cytolytic response (δ>0) sometime after infection [36]. Initial estimates, mostly based on interferon therapy of chronically infected patients, found that the loss rate of infected cells was quite variable with median half-lives of about 7 days [21], [60], [61], corresponding to a probability of death per generation δ = 0.025 (by the same argument using the exponential distribution as above to estimate ρ).

#### θ – probability of a free virus infecting a target cell (if these are available)

In this model, as well as in the standard model of viral infection (i.e., the differential equation model that has been widely used to analyze both primary infection and antiviral treatment [21], [62]), free virus can either be cleared or infect a new cell. In the standard model these processes occur at rates *c* and β*T*, respectively, where *T* is the available target cell density. If we write the differential equations corresponding to the infected cells, *I*, and free virus, *V*, we have
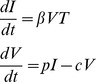
where *p* is the daily viral production rate per infected cell. If we make the common assumption of quasi-steady state, then *I≈(c/p) V* [21], [62]. This essentially means that the viral dynamics are much faster than the infected cell dynamics. From the first equation above, we can now write
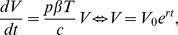
with the initial exponential rate of increase of the virus, *r*, given by *r* = *p*β*T*/*c*. Moreover, in this model the probability of infection is given by θ = β*T*/(*c*+β*T*), because infection (β*T*) is one of two possible events, the other being virion clearance (*c*). We can write this probability of infection in terms of *r* and *p* as θ = *r*/(*r*+*p*). Here, *r* can be measured directly from the rate of exponential increase in viral load observed in the data of each individual. Indeed, the initial rise in viral load is well described by a constant exponential rate of increase, as has been suggested before [30]. In our model, *p*, the daily virus production rate varies over time, because the number of replication complexes in each infected cell is increasing. However, to be consistent with the observed constant rate of increase, *r*, we assumed that *p* = *n*_g_×*RC*_M_×*k*; where *n*_g_ is the number of generations per day (converting the production of viruses per generation of the simulation into the production rate per day), *RC*_M_ is the maximum number of replication complexes, and *k* is the fraction of newly synthesized RNA that is exported as virions. Because we are fitting *k*, this expression for *p* corresponds to a constant effective production rate throughout primary infection that matches the viral load. Substituting this expression for *p* into θ, we have 
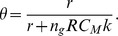


#### *I_ss_* –number of infected cells at steady state

The data of most individuals exhibits a viral plateau a couple of weeks after infection (see also [30]). That is, the virus does not continue to grow exponentially. With our assumption of negligible infected cell death (δ = 0), the logical implication of the observation of this steady state is that the number of infected cells reaches a maximum and is then kept constant at this level, *I_ss_*. When the virus reaches the plateau, *V_ss_*, we can calculate from the standard model and the quasi steady-state assumption [21], [62] that
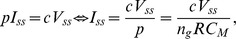
where the daily production of virus per infected cell at the steady state is *p* = *n*_g_×*RC*_M_. Note that at the steady state, each cell has the maximum number of replication complexes, because if it did not then the production per cell would increase further contradicting the assumption of a steady state. Thus, at the steady-state all newly synthesized RNA is exported as new virions, and *k* does not appear in the formula for *I*_ss_. This number represents the number of infected cells at the plateau. For those few cases where we did not observe the viral plateau, because of loss to follow-up, we use for *V*_ss_ the maximum viral load observed. To calculate *I*_ss_, we use *c* = 23 day^−1^, estimated from the rate of decay of HCV RNA in patients treated with an NS5A inhibitor [63].

#### Δ – fraction of lethal mutations

We assume that 40% of all mutations are lethal and that the rest are neutral. The fraction of lethal mutations has been estimated in different viruses using site-directed mutagenesis [48]. For two eukaryotic RNA viruses this fraction was 40% as used here, whereas for an RNA bacteriophage it was 30% [48]. The lethal phenotype can have different causes (from improper folding of the RNA molecule to lack of protease function). Here we will assume that lethal mutations lead to vRNAs that do not contribute to viral load or to make new replication complexes. An alternative view would be that some lethal mutations still allow production of viral particles, but that these are not infectious. In this case, they would be included in viral load measurements, but they would not infect new cells. In our model, this possibility is accounted for by the parameter θ, the fraction of virus that infects new cells.

#### τ – time (in generations) that a cell takes to start producing RNA upon first infection

During stable cell infection and virus production, we have assumed that it takes ∼6 h for a cycle of replication to produce new virions or replication complexes. However, upon initial virus infection, a cell does not produce virus immediately. It goes through an eclipse phase before the first RNAs are produced. Replication in cellular cultures is readily detectable at 24 h, albeit at low levels [4]. Thus, for the baseline scenario, we assume that upon infection cells can start producing vRNAs after a time corresponding to two generations, i.e. 12 h. However, we also investigate the effect of larger values for τ.

#### μ – mutation rate per base per replication (2 copying events)

The mutation rate for HCV has been estimated maximally at μ = 1.2×10^−4^ per base per replication [13]. In our simulations, we choose μ to match the observed profile in the decrease over time of the fraction of sequences without mutations in relation to the putative T/F virus. That is, at each time point for which we have a SGA sample, we calculate for the data and in the simulations the fraction of virus that has not mutated and hence has a genome (segment) still identical to the T/F virus. For each individual and genome segment (quarter or 5′ half), we vary μ in increments of 0.01/*N_b_*, where *N_b_* is the number of nucleotides of the SGA sequence, and find the mutation rate that provides the best fit to the data (i.e., minimizes the sum of the squared residuals). Again, we ran 100 simulations for each value of μ and used the average of those simulations to compare with the data.

## Supporting Information

Text S1 In Text S1 in online supporting information, we show that our results are robust regarding our choices for the maximum number of replication complexes, the initial delay after a cell is infected and before virus is produced and the fraction of lethal mutations. We also present details of the estimation of mutation rate using the frequency of stop codons.
(PDF)
