# Supplementary material for: Quantifying the Diversification of Hepatitis C Virus (HCV) during Primary Infection: Estimates of the In Vivo Mutation Rate
Source: PLoS Pathog. 2012 Aug 23;8(8):e1002881. doi: 10.1371/journal.ppat.1002881 (PMC3426522; doi:10.1371/journal.ppat.1002881)
Supplement: Text S1 — In Text S1 in online supporting information, we show that our results are robust regarding our choices for the maximum number of replication complexes, the initial delay after a cell is infected and before virus is produced and the fraction of lethal mutations. We also present details of the estimation of mutation rate using the frequency of stop codons. (PDF) [file ppat.1002881.s001.pdf]

SUPPORTING INFORMATION

The Mutation Rate Estimates are Robust to Different Choice of Parameters

In the results presented in the main text and our estimation of the mutation rate, we made certain assumptions about model parameters. Here we show that many of these assumptions are not critical or are conservative, in the sense that they lead to higher estimated mutation rates than other parameter possibilities. In each case, we vary only the parameter discussed, and keep all others at the values indicated in the main text and in Table 1.

We show, in Figure S1A, the effect of considering other values for the maximum number of replication complexes, RC_M, within a cell. In the main text we used RC_M=40, but for values above 10 (we show 10, 20, 40 and 80) there is almost no difference, and the result is certainly within the expected variation, in the predicted viral load profile (left panel) and pattern of accumulation of mutations (right panel). Moreover, analysis of the model shows that there is a trade-off between RC_M and the number of infected cells at the plateau (I_{ss}), explaining why different values of RC_M do not change much the predictions of the model. In the case of a mechanism of replication akin to a “stamping machine”, i.e. RC_M=1, we find that the maximum predicted accumulation of mutations is lower than that observed in the data. At the same time, the virus rises faster than seen in the data. This can be understood, because with RC_M=1 all newly synthesized RNA is exported as virions (so k is larger). At the same time, all virions coming from an infected cell have, on average, the same number of mutations, because all virions result from two passages of the polymerase +RNA to –RNA to +RNA. This number of mutations is much less than when multiple replication complexes exist inside that cell.

Next, we varied the initial time it takes for the first RNA to be synthesized upon cell infection (Figure S1B). Again, for a range of realistic values between 12 h and 48 h, there is very little difference in the results predicted by the model. It is only when τ is unrealistically high (here 5 days) that a significant delay is observed. Even in this case, though, we observe only a shift in the profiles of viral load and of accumulation of mutations. If indeed, the delay in first production of RNA were that large, then one would predict that each individual was infected a few days earlier than the infection date on the graph. With such shift of time zero, the model would still fit the data accurately.

We also varied the fraction of mutations that are considered to be lethal, and that lead to non-productive RNA synthesis, Δ, (Figure S1C). For our baseline results we used Δ=0.4. However, if we take this fraction to be 0, 0.2, or 0.8, this does not affect the viral load profile during primary infection (left panel). This may be expected as the RNAs with lethal mutations are a very small fraction of the total number of RNAs produced, since they correspond to 40% of the $\sim 10^{-5}$ mutated RNAs. On the other hand, the effect of different Δ on the expected accumulation of mutations is large (right panel). If the fraction of lethal mutations is less than the original 0.4, then the model predicts more mutations than observed in the data, and to fit the latter we would need even smaller mutation rates than those estimated in the baseline scenario (Table 1). If the fraction of lethal mutations is larger than our original assumption of Δ=0.4, say Δ=0.8 (Figure S1C), then the model predicts a slower accumulation of mutations and to fit the data we would need a larger value for the mutation rate. Still, even in this extreme case, the median estimated mutation is only 5.5×10^{-5} per nucleotide per replication cycle, about twice our estimate for Δ=0.4. However, the value of Δ=0.4 used here is already high, corresponding to the maximum reported in the literature to date [1].

Estimating the Mutation Rate from Stop Codon Frequencies

Classical genetics shows that for a population in mutation-selection balance the frequency, *f*, of single point mutations with selection disadvantage *s* is given by $f=\mu/s$, where μ is the mutation rate. For lethal mutations, s=1, and the frequency of these mutations is simply $f=\mu$, since they must have been generated at the last replication cycle. Cuevas *et al.* [2] proposed using this approach to estimate the mutation rate of HCV, using non-sense mutations, i.e. stop codons (UAA, UAG, UGA) as a proxy for lethal mutations.

There are 18 codons that upon mutation may generate a stop codon. These are: U**U**A, UUG, U**C**A, UCG, UA**U**, UA**C**, UGU, UGC, UGG, CAA, CAG, CGA, AAA, AAG, AGA, GAA, GAG, GGA. Following the notation in [2], in each codon we underline the nucleotide that is the mutation target, for a total of 19 non-sense mutation targets – NSMT (note that UGG has two targets). A priori, each of these targets can mutate to any of the other 3 nucleotides, but in most cases only the mutation to one of those 3 will generate a stop codon (for example, UGU -> UGA). In a few cases the underlined nucleotide can mutate to 2 of the 3 possibilities and generate a stop codon, and we use boldface to indicate such cases (for example, UAU -> UAA or UAG). We can now count in the data the number of NSMT (*M*), the number of stop codons

(N), and the parental NSMT of each stop codon, such that we have N_1 stop codons generated by mutation of non-boldface, underlined nucleotides and N_2 stop codons originated by mutation of boldface, underlined nucleotides.

Cuevas *et al.* [2] then used the following reasoning. Let us assume that there are M_1 codons (underlined), for which a single nucleotide substitution leads to a stop codon; M_2 codons (underlined, boldface), for which two possible mutations lead to stop codons, with $M= M_1 + M_2$; and that the mutation rate per nucleotide per replication cycle is μ. Then in one replication cycle, we can write $N_1 = (M_1 + N_1)\mu\frac{1}{3}$ and $N_2 = (M_2 + N_2)\mu\frac{2}{3}$, where the 1/3 comes from the fact that only one of the three possible nucleotide changes leads to a stop codon, and similarly for the 2/3 when two of the three possible changes lead to a stop codon. Note also that we added N_1 to M_1 and N_2 to M_2, because the codons that did mutate were also targets before this replication cycle, and since they mutated they were not counted in M_1 or M_2. However, because N_1 and N_2 are about 10^5-fold smaller than M_1 and M_2, respectively, to simplify we can also neglect the N_1 added to M_1 and the N_2 added to M_2. Upon rearrangement of the two expressions, this simplification allows us to use the same formula as in Cuevas *et al.* [2]:

$$\begin{cases} M_1\mu = 3N_1 \\ M_2\mu = \frac{3}{2}N_2 \end{cases} \Rightarrow M_1\mu + M_2\mu = 3N_1 + \frac{3}{2}N_2 \Leftrightarrow \mu = \frac{1}{M}\left(3N_1 + \frac{3}{2}N_2\right)$$

By counting each stop codon appropriately and the total number of NSMT, one can use this formula to directly estimate the mutation rate, as we do in the text.

It is important to note that each of the two expressions alone would allow us to estimate μ (i.e., $\mu=\mu_1\equiv 3N_1/M_1$ or $\mu=\mu_2\equiv(3/2)(N_2/M_2)$). By using both together, we are in a way averaging those two individual mutations estimates. We now propose a slightly different way to estimate μ (i.e., in effect a different linear combination of those two expressions), by summing the expressions defining N_1 and N_2 to obtain

$$N_1 + N_2 = \frac{1}{3}\left(M_1\mu + 2M_2\mu\right) \Leftrightarrow \mu = \frac{3(N_1 + N_2)}{M_1 + 2M_2}.$$

This expression for μ has a simple interpretation. We divide the actual number of stop codons observed (N_1+N_2) by the total number of possible mutations leading to stop codons (M_1+2M_2). The factor 3 corrects for the fact that for each stop codon mutation observed, it is equally likely that the original nucleotide mutated to a non-stop codon nucleotide, which we did not count [3]. The two expressions that we derived for μ give very similar estimates as shown in the main text, and we can show that in the limit of infinite data they are the same. Further, as we show below, the second expression is statistically a more efficient estimator, because it has a smaller sampling variance.

Since both μ_1 and μ_2 are independent estimates of μ, we can use the weighted average $\gamma\mu_1+(1-\gamma)\mu_2$ for any weight γ ($0<\gamma<1$) as an estimator of μ. The sampling variances of the two starting estimators are, however, different in general, and hence, the sampling variance of these averages depends on α. In particular, since μ is small, we can ignore the possibility of multiple mutations within a single codon, and the distributions of N_1 and N_2 are binomial with rates $\mu/3$ and $2\mu/3$ and sizes M_1 and M_2, respectively. The variances of N_1 and N_2 are then $M_1(\mu/3)(1- \mu/3)$ and $M_2(2\mu/3)(1- 2\mu/3)$, respectively. However, from the definitions of μ_1 and μ_2, $N_1= M_1\,\mu_1/3$ and $N_2 = 2M_2\mu_2/3$, thus the variance (*Var*) of $Var(N_1) = Var(M_1\,\mu_1/3)= (M_1/3)^2\ Var(\mu_1)$ and similarly $Var(N_2)=(2M_2/3)^2\ Var(\mu_2)$. The resulting sampling variances of μ_1 and μ_2 are then given by $3\mu_1(1-\mu_1/3)/M_1\approx3\mu_1/M_1$ and $3\mu_2(1-2\mu_2/3)/2M_2\approx3\mu_2/2M_2$, respectively. The sampling variance of the weighted average is then $3[\gamma^2/M_1+(1-\gamma)^2/2M_2]\mu$ (where we have used μ in place μ_1 and μ_2), which is minimized when $\gamma= M_1/(M_1+2M_2)$. The use of this optimal weight for the weighted average of the two estimates μ_1 and μ_2 leads to our second expression above for estimating μ. This result can be trivially generalized to an arbitrary number of independent binomial processes with rates proportional to a single parameter, μ, to be estimated: when the rates are small, the minimal variance estimator is given by the total number of observed events divided by the expected number per unit μ.

We note that both expressions for μ are still an approximation, because they assume that all types of substitutions are equally likely, which is not correct. For example, transitions are preferred over transversions, and indeed we found that this bias is 18 to 1 (α=18) in our data set, when corrected for available sites [4]. One way to implement this correction in the last formula for μ is to count the possible mutations according to whether they represent transitions (T) or transversions (V), so that $M_1=M_1^T+M_1^V$ and $M_2=M_2^T+M_2^V$. The codons that can mutate by transition to a stop codon are: CGA, CAA, CAG, and UGG; all others require a transversion. Then one uses the bias ratio (α) to weigh the mutations, such that we have a new effective number of allowed mutations $M_1^*=(\alpha M_1^T+M_1^V)/\alpha$ and

$2M_2^*=((1+\alpha)M_2^T+2M_2^V)/\alpha$. In the same way, the factor 3, which assumes that any nucleotide substitution is equally likely, has to be replaced by $(\alpha+2)/\alpha$, since a nucleotide can mutate by a transition or two transversions. Thus, we have

$$\mu=\frac{\left((\alpha+2)/\alpha\right)\left(N_1+N_2\right)}{M_1^*+2M_2^*},$$

and if $\alpha=1$, i.e. transitions occur at the same rate as transversions, one recovers the previous formula for μ. In the other limit, if transversions were not allowed, then α is infinite, and $\mu=(N_1+N_2)/(M_1^T+M_2^T)$. In the same spirit, other corrections, such as codon usage or matrices for favored mutations, could also be accounted for.

In Table S1, we present the details of the stop codons found in our data set. In Table S2, we show the NSMT in the data set. With this data, we can calculate the mutation rates presented in the text. Thus, if instead of assuming that transitions and transversions occur at the same rate, we assume the limit case of allowing only transitions, then we obtain $\mu= 3.4 \times 10^{-5}$ (95% CI [1.6,6.3]), just slightly larger than the 2.8×10^{-5} mutations/nucleotide/replication shown in the main text.

Table S1. Stop codons found in the acute infection data set (1617 half genomes, 556 1st quarter and 363 2nd quarter genomes). Thirteen stop codons were observed but 4, bolded in the table, were at the same site and apparently from a single replication complex so they were counted only once.

Patient ID	Sequence ID	Codon	From	To	Mutation	Type
**P10051**	**10051.11.2B8**	**733**	**GAG**	**UAG**	**G->U**	**Transversion**
**P10051**	**10051.11.2B9**	**733**	**GAG**	**UAG**	**G->U**	**Transversion**
**P10051**	**10051.14.2C2**	**733**	**GAG**	**UAG**	**G->U**	**Transversion**
**P10051**	**10051.14.2C3**	**733**	**GAG**	**UAG**	**G->U**	**Transversion**
P106889	106889.5.02D16	66	UGG	UAG	G->A	Transition
P10003	10003_07B8	304	UGG	UAG	G->A	Transition
P10003	10003_07NC18	1196	CAG	UAG	C->U	Transition
P10012	10012.06.5Q1.TB9	700	UGG	UGA	G->A	Transition
P10017	10017.10.E13	702	UAC	UAG	C->G	Transversion
P10017	10017.14.C4	388	CAA	UAA	C->U	Transition
P10021	10021.14.TD2	759	UGG	UGA	G->A	Transition
P10021	10021.08.5Q2.B12	110	UGG	UAG	G->A	Transition
P10029	10029.08.5Q1.2B27	621	GAG	UAG	G->U	Transversion

Table S2. Number of NSMT in our data set. We indicate in bold those codons that can mutate to a stop codon by a transition, the others require a transversion. Note that UGG should be counted twice, as explained in the text.

**CGA**	**CAA**	**CAG**	**UGG**	UUA	AGA	UCA	GAA	AAA
15073	38590	63846	87519	12869	19716	21173	21334	25591
UCG	UGU	GGA	UAU	AAG	UUG	GAG	UGC	UAC
29033	41344	42651	43488	59069	60905	66622	79750	82243

Figure S1. Changes in viral load and mutation profile predicted by the model for different values of the (A) maximum number of replication complexes (RC_M) in an infected cell, (B) time until the production of the first RNA (τ), and (C) proportion of lethal mutations (Δ).

A

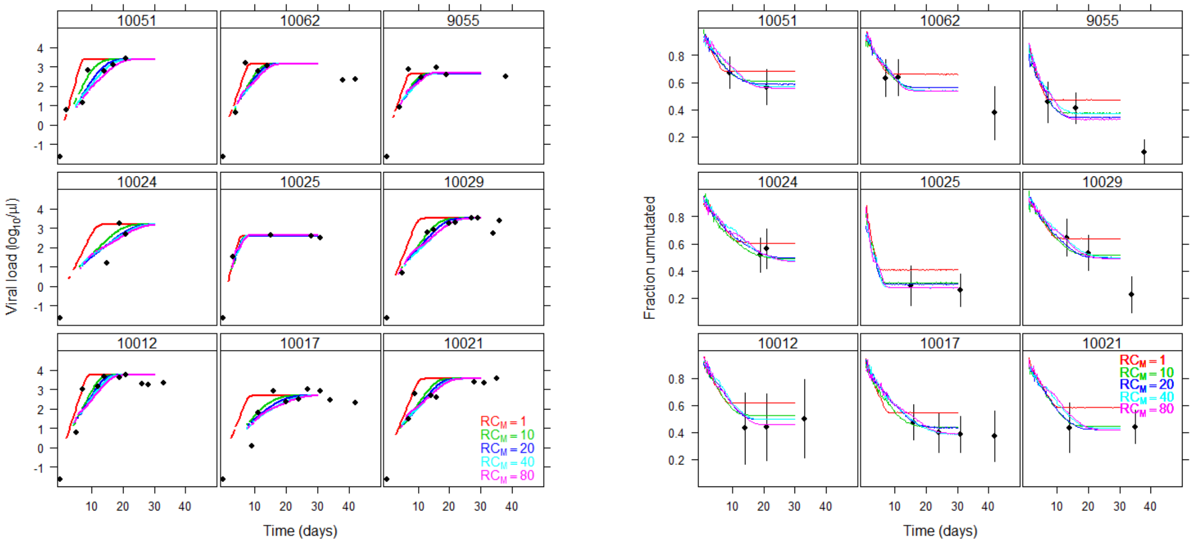

B

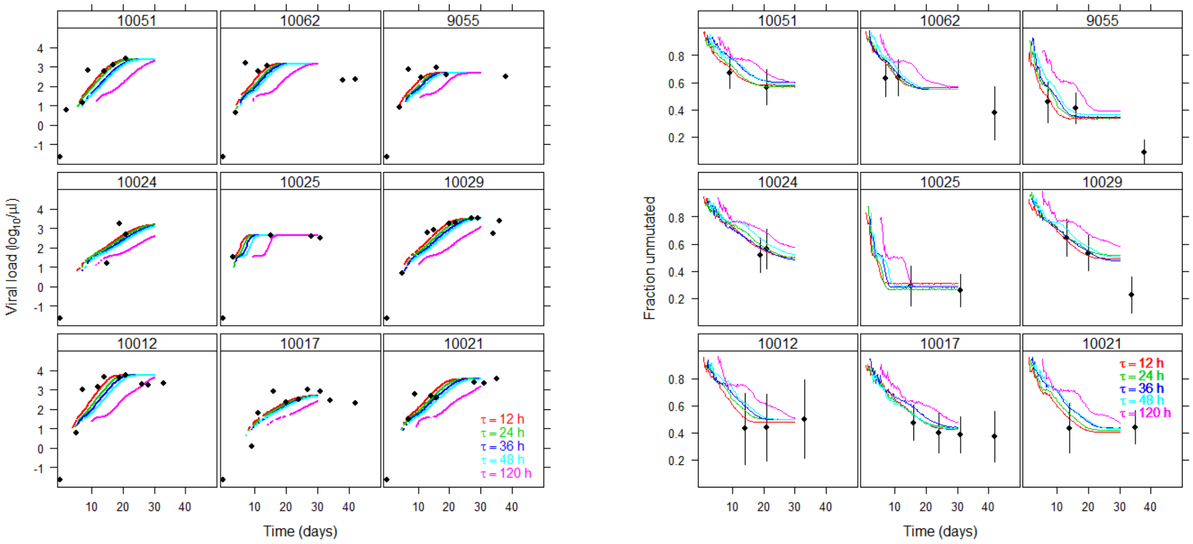

C

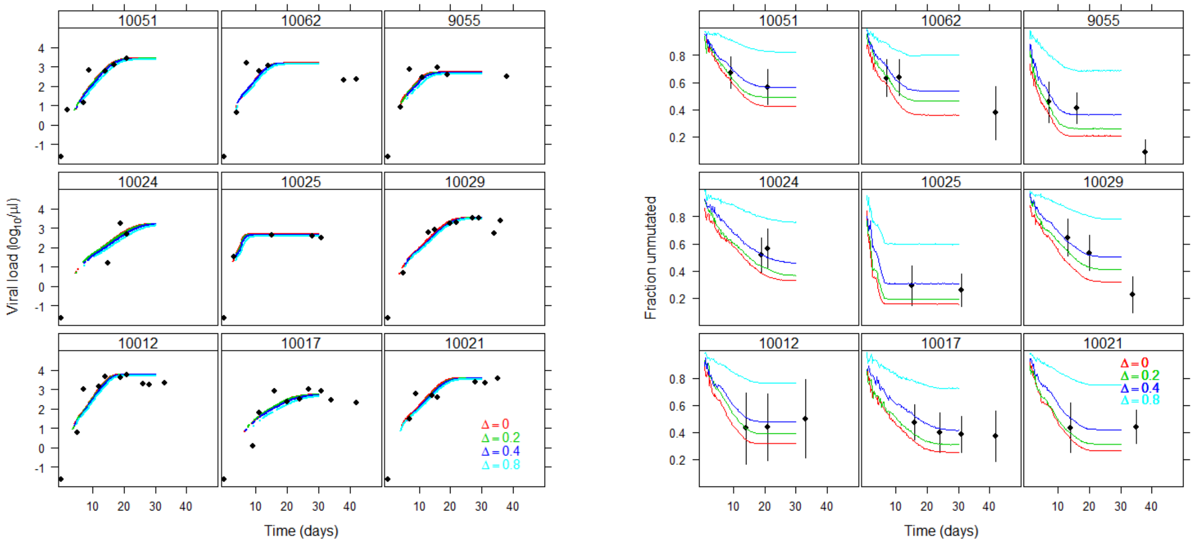

REFERENCES:

1. Sanjuan R (2010) Mutational fitness effects in RNA and single-stranded DNA viruses: common patterns revealed by site-directed mutagenesis studies. Philos Trans R Soc Lond B Biol Sci 365: 1975-1982.

2. Cuevas JM, Gonzalez-Candelas F, Moya A, Sanjuan R (2009) Effect of ribavirin on the mutation rate and spectrum of hepatitis C virus in vivo. J Virol 83: 5760-5764.

3. Drake JW, Holland JJ (1999) Mutation rates among RNA viruses. Proc Natl Acad Sci U S A 96: 13910-13913.

4. Li H, Stoddard MB, Wang S, Parrish EH, Learn GH, et al. (2012) Elucidation of hepatitis C virus transmission and early diversification by single genome sequencing. PLoS Pathogens in press.
